# Electroporated Antigen-Encoding mRNA Is Not a Danger Signal to Human Mature Monocyte-Derived Dendritic Cells

**DOI:** 10.1155/2015/952184

**Published:** 2015-12-28

**Authors:** Stefanie Hoyer, Kerstin F. Gerer, Isabell A. Pfeiffer, Sabrina Prommersberger, Sandra Höfflin, Tanushree Jaitly, Luca Beltrame, Duccio Cavalieri, Gerold Schuler, Julio Vera, Niels Schaft, Jan Dörrie

**Affiliations:** ^1^Department of Dermatology, Universitätsklinikum Erlangen, Erlangen, Germany; ^2^Department of Genetics, Friedrich-Alexander-Universität Erlangen-Nürnberg, Erlangen, Germany; ^3^Department of Oncology, Istituto di Ricerche Farmacologiche Mario Negri, Milan, Italy; ^4^Department of Neurosciences, Psychology, Drug Research and Child Health, University of Florence, Florence, Italy

## Abstract

For therapeutic cancer vaccination, the adoptive transfer of mRNA-electroporated dendritic cells (DCs) is frequently performed, usually with monocyte-derived, cytokine-matured DCs (moDCs). However, DCs are rich in danger-sensing receptors which could recognize the exogenously delivered mRNA and induce DC activation, hence influencing the DCs' immunogenicity. Therefore, we examined whether electroporation of mRNA with a proper cap and a poly-A tail of at least 64 adenosines had any influence on cocktail-matured moDCs. We used 16 different RNAs, encoding tumor antigens (MelanA, NRAS, BRAF, GNAQ, GNA11, and WT1), and variants thereof. None of those RNAs induced changes in the expression of CD25, CD40, CD83, CD86, and CD70 or the secretion of the cytokines IL-8, IL-6, and TNF*α* of more than 1.5-fold compared to the control condition, while an mRNA encoding an NF-*κ*B-activation protein as positive control induced massive secretion of the cytokines. To determine whether mRNA electroporation had any effect on the whole transcriptome of the DCs, we performed microarray analyses of DCs of 6 different donors. None of 60,000 probes was significantly different between mock-electroporated DCs and MelanA-transfected DCs. Hence, we conclude that no transcriptional programs were induced within cocktail-matured DCs by electroporation of single tumor-antigen-encoding mRNAs.

## 1. Introduction

During the last decade, immunotherapy has evolved as a new pillar of cancer treatment [1]. Therapeutic vaccination with dendritic cells (DCs) is a safe and well-established strategy [2–4]. A deeper understanding of DC maturation and activation together with efficient, GMP-compliant and reproducible antigen- (Ag-) loading strategies is the key to success. One technology that has proven suitable in this context is mRNA transfection [3, 5, 6], which can be utilized, on the one hand, to load mature DCs with tumor antigen [7–9], and on the other hand, to deliver maturation and activation signals to the DCs. The latter is usually achieved by using mRNA that encodes DC-activating proteins, like constitutively active inhibitor of kappa B kinase (IKK) [10] or CD40L, alone [11], or combined with a constitutively active TLR [12].

However, since DCs comprise a whole battery of nucleic acid receptors on their surface, in their endosomes, and in their cytoplasm [13, 14], the transfected mRNA itself, independently from the encoded protein, may deliver a maturation signal. Single-stranded (ss)RNA was reported to activate TLR7 and TLR8 on DCs [15–17] and TLR3 can be activated by short double-strand stretches in exogenous mRNA [18, 19]. RIG-I-like receptors (RLR) recognize various viral RNA species in the cytoplasm [20] and may be capable of sensing transfected RNA as well. Bacterial RNA is a potent DC maturation stimulus, but the specific receptors are yet unknown [21].

In the design of clinical vaccination protocols, it is, however, pivotal to know which maturation program is induced in the DCs and whether and how any additional maturation stimulus might distort the intended mature phenotype of the vaccine DCs. When DCs (usually monocyte-derived ones) are generated, matured, and Ag-loaded for clinical application, one requires a well-defined product, and any remaining insecurity about any factors that influence the phenotype of the DCs should be resolved.

Hence, we took the effort to carefully compare monocyte-derived cytokine-matured DCs that were electroporated without RNA with DCs that were electroporated with various* in vitro*-transcribed mRNAs. We analyzed the DCs' phenotype and cytokine secretion and, for mRNA, encoding the tumor antigen MelanA, a transcriptome analysis was performed, to detect if any of these features would be changed by the introduced mRNA.

## 2. Materials and Methods

### 2.1. Cells and Reagents

Monocyte-derived dendritic cells (DCs) were generated from blood, obtained from healthy donors following informed consent and approval by the institutional review board as described before [9]. PBMCs were purified by density centrifugation, and monocytes were separated from the nonadherent fraction (NAF) by plastic adherence and differentiated to DCs over 6 days in DC medium (RPMI 1640 (Lonza, Verviers, Belgium) containing 1% heat-inactivated autologous plasma, 2 mM L-glutamine (Lonza), and 20 mg/L gentamicin (PAA, Pasching, Austria)) with GM-CSF (800 IU/mL; CellGenix, Freiburg, Germany, PeproTech, Hamburg, Germany, and Miltenyi Biotec, Bergisch Gladbach, Germany) and IL-4 (250 IU/mL; CellGenix, PeproTech, and Miltenyi Biotec) in the absence of fetal calf serum, as described before [9]. DCs were matured (mDCs) on day 6 for 24 h with 200 IU/mL IL-1*β* (CellGenix), 1000 U/mL IL-6 (CellGenix), 10 ng/mL TNF*α* (Beromun, Boehringer Ingelheim Pharma, Germany), and 1 *μ*g/mL PGE_2_ (Pfizer, Zurich, Switzerland). mDCs were used for electroporation with mRNA after maturation.

### 2.2. *In Vitro* RNA Transcription and Electroporation of DCs

*In vitro* transcription of mRNA from pGEM4Z64A vectors was performed as described previously [9] with Life Technologies mMESSAGE mMACHINE T7 ULTRA kits according to the manufacturer's instructions. DCs were electroporated with different mRNAs (Table 1) as described in [10, 22]. As a control, mDCs were electroporated without mRNA.

### 2.3. Cell Surface Marker Analysis

mDCs were electroporated as described above, incubated in DC medium at 37°C in a humidified incubator, and harvested 24 h after electroporation. The expression of distinct markers was analyzed by flow cytometry. For the determination of surface marker expression, the following antibodies and their respective isotype controls were used: IgG1-PE, anti-CD25-PE, anti-CD40-PE, anti-CD70-PE, anti-CD80-PE, anti-CD83-PE, anti-CD86-PE (all from BD), and IgG3-PE (eBioscience). Seventy-five to one hundred thousand cells were incubated with antibody for 30 minutes at 4°C in FACS solution, consisting of PBS supplemented with 1% FCS (PAA, GE healthcare) and 0.02% sodium azide (Merck). The cells were then washed once with FACS solution and immunofluorescence was measured using a FACScan cytofluorometer equipped with CellQuest software (BD Biosciences). mDCs were gated on in the forward and side scatter channels and the mean fluorescence intensities (MFIs) were measured. Specific MFI was calculated by subtraction of the MFI of the isotype control.

### 2.4. Cytokine Secretion Analysis

mDCs were electroporated as described above and were incubated in DC medium at 37°C in a humidified incubator, and supernatants were taken 24 h after electroporation. Cytokine concentrations were analyzed with an Inflammation Cytometric Bead Array (BD, Heidelberg, Germany) following the manufacturer's instructions.

### 2.5. Statistical Analysis

We performed a 1-way ANOVA with multiple comparison test (according to Dunnet/Tukey) with a confidence level of 0.05 using GraphPad Prism V6.02 to determine statistically significant differences for the surface staining data (Figure 2) with the unadjusted mean fluorescence intensities, the percentage values of positive cells, and the cytokine concentrations (Figures 3 and 4). For the data in Figures 2 and 3, the multicomparison was performed according to Dunnet against the mock condition. For the data in Figure 4, all conditions were compared to each other according to Tukey.

### 2.6. Cryoconservation of Cell Pellets

mDCs were electroporated as described above, were incubated in DC medium at 37°C in a humidified incubator, and were harvested 4 h after electroporation. One to two hundred thousand cells were centrifuged for 10 min at 10.000 rpm at 4°C and the supernatant was removed. The cell pellet was frozen and stored in liquid nitrogen until microarray analysis.

### 2.7. Microarray Analysis

Cryoconserved electroporated mDCs were sent to Miltenyi Biotec for microarray analysis. Cells were lysed, mRNA was isolated and reverse-transcribed to cDNA, the cDNA was amplified, and Cy3 was labeled and then hybridized to Agilent Whole Human Genome Oligo Microarrays (8 × 60 K). Fluorescence signals of the hybridized Agilent Microarrays were detected using Agilent's Microarray Scanner System (Agilent Technologies). The Agilent Feature Extraction Software (FES) was used to read out and process the microarray image files. The resulting text files produced by FES were then processed for quality control, removing control probes and probes flagged as unreliable by the scanning software. The raw data underwent background correction to eliminate background noise and local fluctuations. To this end, the normal-exponential convolution method was used (Normexp). Next, the data were normalized to correct chip-related variations in the signal intensity (e.g., labeling and hybridization inefficiencies). To this end, the quantile method with offset = 16 was applied to the data.

Unadjusted *p* values were calculated with Student's *t*-test and the Benjamini Hochberg method for False Discovery Rate was used to adjust the *p* value and find differentially expressed genes. Data processing and analysis were performed in the software R computing environment (version 3.0.2) using the Bioconductor (version 3.1) package “limma” (linear models for microarray and RNA-seq data) described in [23].

## 3. Results

### 3.1. mRNA Electroporation into Human Cocktail-Matured, Monocyte-Derived DCs Results in a High Transfection Efficiency

**Figure 1:**
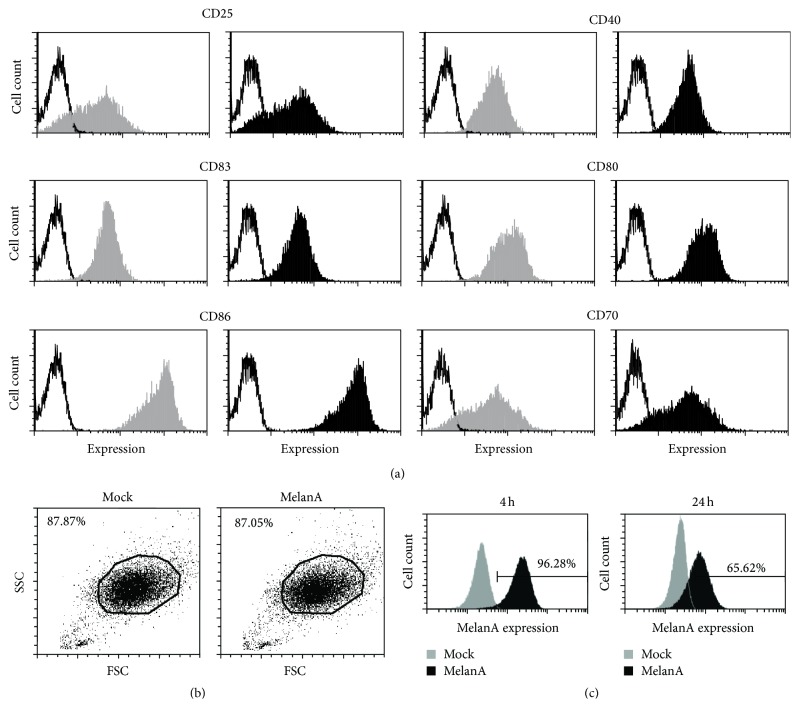
MelanA is expressed in cocktail-matured, monocyte-derived DCs after mRNA electroporation. DCs were either electroporated without mRNA (mock; gray histogram) or with mRNA encoding the tumor antigen MelanA (MelanA; black histogram). (a) Surface marker expression of CD25, CD40, CD83, CD86, CD70, and CD80 on mock-electroporated and MelanA-RNA-electroporated DCs 24 h after electroporation is shown (black lines; respective isotype controls). One representative of ≥4 experiments is shown. (b) Gating of mock-electroporated (Mock) or MelanA-RNA-electroporated (MelanA) DCs was performed according to forward and side scatter. (c) Four and twenty-four hours after electroporation, the intracellular MelanA expression was determined by flow cytometry. The percentage of positive cells is indicated. One representative of >10 experiments is shown.

To formally show that mRNA electroporation results in protein expression in the cocktail-matured, monocyte-derived DCs, we generated these DCs and electroporated them either without RNA (mock) or with RNA encoding the tumor antigen MelanA (Figure 1). These DCs were produced by a highly standardized and validated process, which is approved for DC generation for clinical applications [24]. Hence, the product is very well known considering the phenotype of the DCs. Therefore, we did not include a typical DC-specific marker in these experiments but rather focused on maturation and activation markers on these DCs. The DCs displayed a mature phenotype, which was not altered by transfection with MelanA-encoding mRNA (Figures 1(a) and 1(b)). Four and twenty-four hours after electroporation, the intracellular MelanA expression was determined by flow cytometry. As shown in Figure 1(c), MelanA expression was detected at both time-points. The transfection efficiency at 4 h was >95% (Figure 1(c); left panel). Due to the transiency of mRNA transfection, the MelanA expression had decreased at the 24 h time-point (Figure 1(c); right panel). These data show that the electroporated mRNA enters the cytoplasm of the vast majority of the mature DCs very efficiently.

### 3.2. mRNA Electroporation into Human Cocktail-Matured, Monocyte-Derived DCs Has No Influence on the Phenotype of These Cells

**Figure 2:**
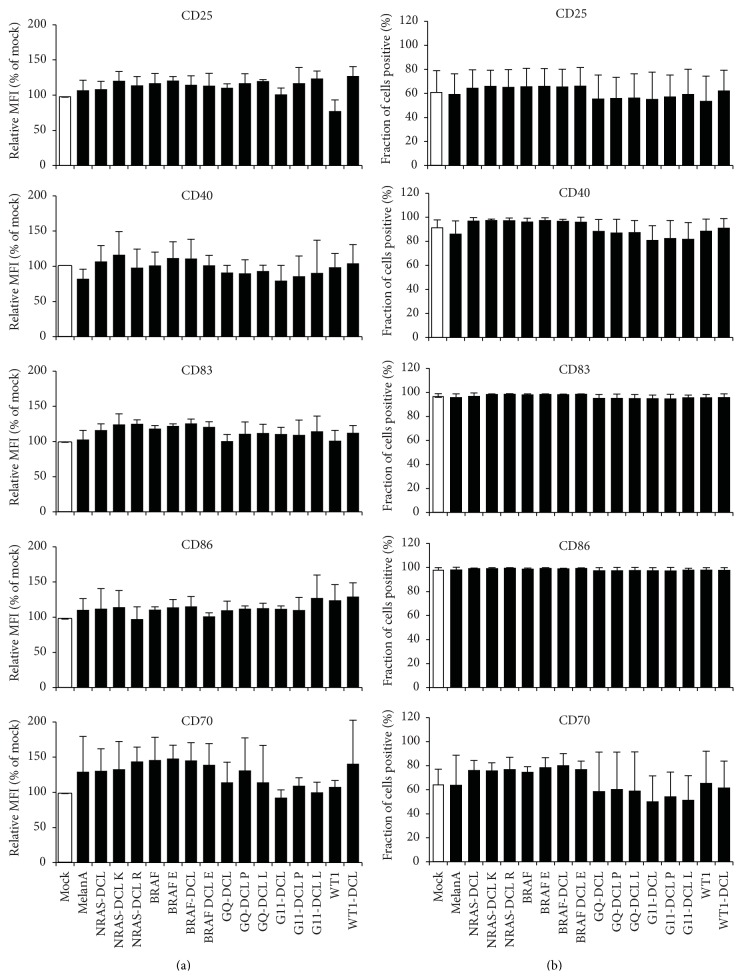
No difference in phenotype between mock- and mRNA-electroporated DCs. DCs were either electroporated without RNA (Mock) or a panel of 16 RNAs encoding different tumor antigens, either mutated or not and either linked to the lysosomal targeting signal DC-LAMP or not (see Table 1). Twenty-four hours after electroporation, the surface expression of CD25, CD40, CD86, CD70, and CD83 was determined by flow cytometry. Surface marker expression of mock-electroporated DCs was put at 100% and marker expression after electroporation of the mRNAs was put in relation to that (a), or percent positive cells are shown (b). Shown are averages of at least 3 independent experiments. Error bars indicate the standard deviation (SD). According to a 1-way ANOVA with multiple comparison test, no statistically significant differences were present within the surface staining data (*p* > 0.05).

As it was suggested that introduction of mRNA could trigger intracellular TLRs or other receptors [15–21], we examined whether mRNA electroporation has an influence on the phenotype of cocktail-matured, monocyte-derived DCs. We electroporated these DCs either without RNA (Figure 2; mock) or with a panel of 16 different RNAs encoding tumor antigens, or parts thereof (MelanA, NRAS, BRAF, GNAQ, GNA11, and WT1), either mutated or not, and either linked to the lysosomal targeting signal DC-LAMP or not (Table 1). Twenty-four hours after electroporation, the surface expression of CD25, CD40, CD86, CD70, and CD83 was determined by flow cytometry. When looking at mean fluorescence intensities (MFIs), electroporations with the 16 different RNAs encoding different tumor antigens resulted in differences in cell surface marker expression of less than 1.5-fold compared to mock-electroporated DCs (Figure 2(a)). No large differences in expression of these markers were observed when looking at percent positive cells (Figure 2(b)). According to a 1-way ANOVA with multiple comparison test, no statistically significant differences were present within the surface staining data (*p* > 0.05). We did not measure expression of MHC-class II, as we had observed before that there are no big changes on human monocyte-derived DCs, even after activation of NF-*κ*B (data not shown).

From these data, we can conclude that the introduction of mRNA into human cocktail-matured, monocyte-derived DCs by electroporation did not result in a relevant change of the phenotype of these cells.

### 3.3. mRNA Electroporation into Human Cocktail-Matured, Monocyte-Derived DCs Has No Influence on the Cytokine Secretion by These Cells

**Figure 3:**
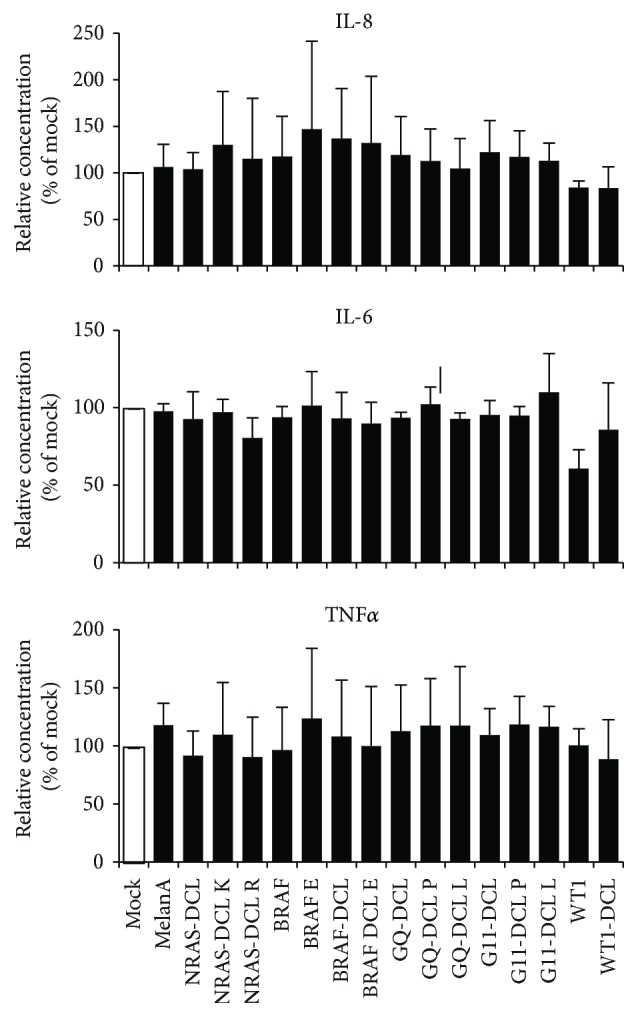
No difference in cytokine secretion between mock- and MelanA-RNA-electroporated DC. DCs were electroporated either without RNA (Mock) or with a panel of 16 RNAs encoding different tumor antigens, either mutated or not, and either linked to the lysosomal targeting signal DC-LAMP or not (see Table 1). Twenty-four hours after electroporation, supernatants of the cells were taken and the cytokine secretion by the cells was determined in a cytometric bead array. Cytokine secretion of mock-electroporated DCs was defined as 100% and concentrations after electroporation of the mRNAs were put in relation to that. Shown are averages of at least 3 independent experiments. Error bars indicate the standard deviation (SD). According to a 1-way ANOVA with multiple comparison test, no statistically significant differences were present within the cytokine secretion data (*p* > 0.05).

Next, we investigated whether mRNA electroporation has an influence on the cytokine secretion of cocktail-matured, monocyte-derived DCs. We electroporated DCs either without RNA (Figure 3; Mock) or with the 16 different RNAs encoding tumor antigens and harvested the supernatants of the cells 24 h after electroporation to determine the cytokine secretion in a cytometric bead array. Within 24 h after electroporation, the DCs hardly any IL-1*β*, IL-10, and IL-12p70 (data not shown) but produced measurable quantities of IL-8, IL-6, and TNF*α* (Figure 3). However, mock-electroporated DCs also secreted these cytokines, and the electroporations with the 16 different RNAs, encoding different tumor antigens, resulted in a difference in cytokine secretion of maximum 1.5-fold compared to mock-electroporated DCs (Figure 3). According to a 1-way ANOVA with multiple comparison test, no statistically significant differences were present within the cytokine secretion data (*p* > 0.05).

These data show that the electroporated mRNAs also had no relevant influence on cytokine secretion by human cocktail-matured, monocyte-derived DCs.

**Figure 4:**
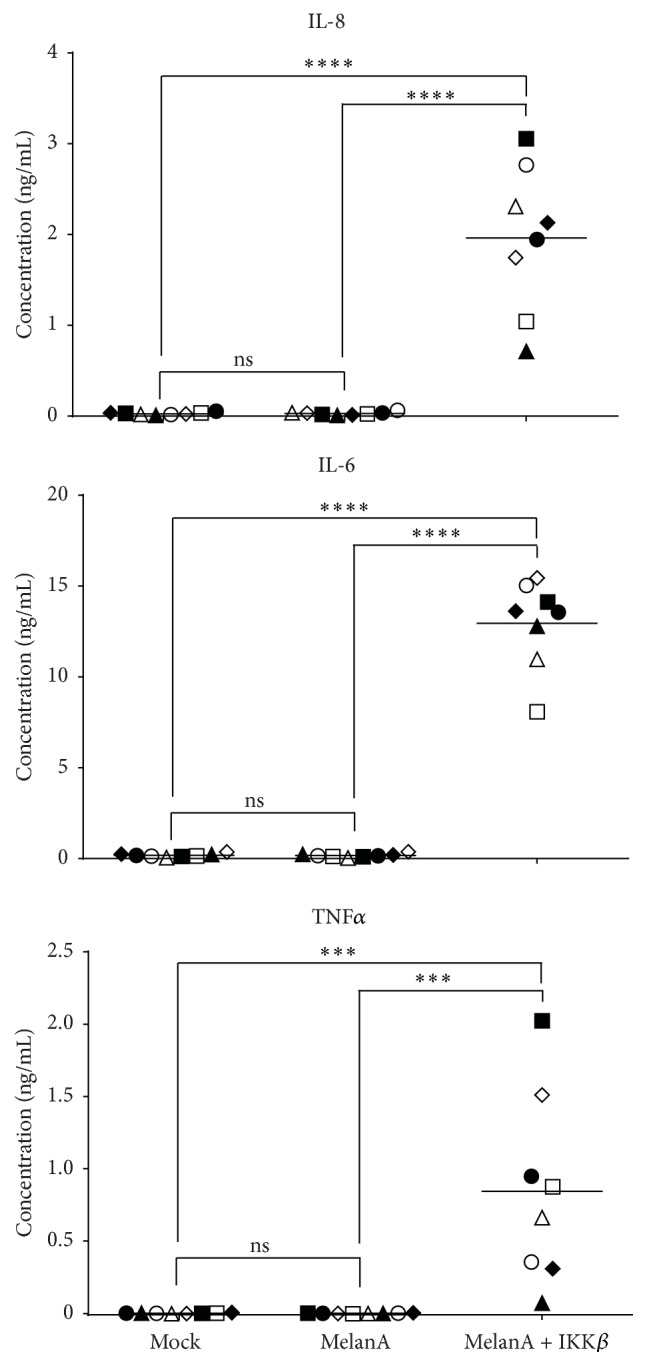
Massive cytokine secretion after electroporation with mRNA encoding a DC-activating protein. DCs were electroporated either without RNA (Mock) or with RNA encoding the tumor antigen MelanA (MelanA) or MelanA and constitutively active stabilized mutated IKK*β*. Twenty-four hours after electroporation, supernatants of the cells were taken and the cytokine secretion by the cells was determined in a cytometric bead array. The cytokine concentrations in the supernatants are depicted. Each symbol represents an individual donor, tested in an independent experiment (*n* = 8). The horizontal bars show the average values. *p* values were calculated by the 1-way ANOVA with multiple comparison test (according to Tukey) with a confidence level of 0.05; ns: *p* > 0.05, ^*∗∗∗*^ *p* ≤ 0.001, and ^*∗∗∗∗*^ *p* ≤ 0.0001.

Due to the fact that the secretion of IL-8, IL-6, and TNF*α* was clear but at low quantities, we wanted to formally prove that our DCs were able to produce these cytokines at higher quantities, when properly activated under similar conditions. Therefore, we electroporated the mature DCs either without RNA or with RNAs encoding MelanA combined or not with RNA encoding a constitutively active form of IKK*β*, which is, on the protein level, able to activate the NF-*κ*B pathway in the DCs [10]. Indeed, we saw that transfection with constitutively active IKK*β* resulted in high IL-8, IL-6, and TNF*α* secretion (Figure 4), proving that our DCs can produce these cytokines at high quantities. In addition, there was no difference between the cytokine secretion by mock-transfected and MelanA-transfected DCs (Figure 4), again showing that mRNA transfection* per se* did not induce cytokine production in DCs. The 1-way ANOVA with multiple comparison test showed that the IKK*β*-transfected DCs were highly significantly different from the mock and MelanA conditions but that the mock and MelanA conditions were not.

### 3.4. Microarray Analysis Reveals No Differentially Expressed Genes between Mock- and MelanA-Transfected DCs

**Figure 5:**
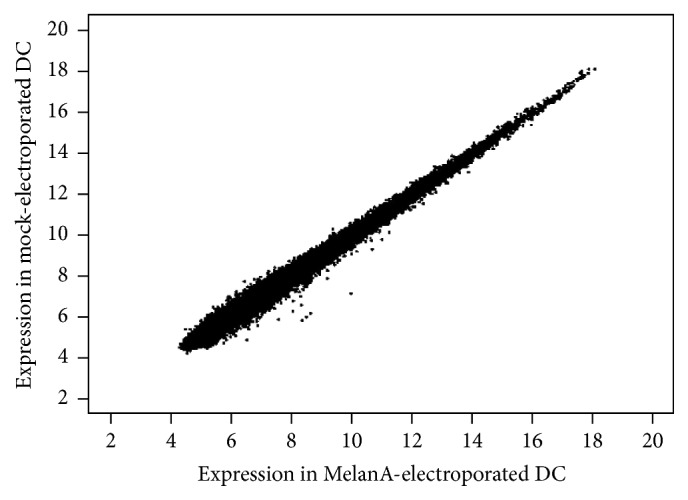
No difference in microarray analysis between mock- and MelanA-RNA-electroporated DCs. Scatter plot of the probe set expression levels for MelanA-RNA-electroporated DCs versus Mock-electroporated DCs samples. Data from 6 samples per experimental condition were processed for background correction and normalization. In the figure, the average probe intensity for each experimental condition is visualized.

Although we found no obvious differences in the expression of a handful of surface markers and the secretion of half a dozen of cytokines upon mRNA electroporation, we still could not exclude that the exogenous mRNA would induce signaling within the DCs, which, by chance, would regulate other target genes and modulate the expression of other factors. To explore in more detail whether mRNA electroporation has any effect on the transcriptome of the DCs, we performed GeneChip microarray analyses with matured DCs (mDCs) of 6 independent donors, which had either been mock-electroporated or electroporated with MelanA RNA. DCs were harvested and frozen 4 h after electroporation, and samples were hybridized to Agilent Whole Human Genome Oligo Microarrays (8 × 60 K). Fluorescence signals of the hybridized Agilent Microarrays were determined, preprocessed, and normalized. Afterwards, differentially expressed genes were calculated and significance was examined by Student's *t*-test and subsequent adjustment using the Benjamini Hochberg method for False Discovery Rate. These calculations were performed using the limma (linear models for microarray and RNA-seq data) software package for the Bioconductor R computing environment [23] (see Section 2). The expression levels of all the microarray probes are compared directly in Figure 5 to depict the degree of difference between the two sample groups. The scatter plot shows that the individual values are close to the identity function (Figure 5), except for a small number of outliers, of which none was significant (Figure 5). Indeed, none of the probes showed a significant difference between mock-electroporated DCs and MelanA-electroporated DCs, and the adjusted *p* values were all above 0.99995, indicating that no differentially expressed genes (DEGs) were present. This suggests that the difference at the transcriptional level between mock-electroporated DCs and MelanA-electroporated DCs is negligible.

## 4. Discussion

In this study, we have shown that electroporation of antigen-encoding RNA into matured monocyte-derived dendritic cells (moDCs) had no influence on the phenotype of these DCs and on the cytokine secretion by these DCs and even that there was no influence of the RNA on the transcriptome of the DCs. This is pivotal information for the use of mRNA-electroporated moDCs in a clinical setting, since it is necessary to generate vaccines of consistent quality by a stable production process, no matter what antigen-encoding RNA is used for electroporation, and it shows that our matured moDCs are of a robust phenotype. Since we [9] and others [8, 11, 25] observed that DCs, which were antigen-electroporated after cytokine-maturation, seemed to perform better, we limited our analysis to DCs matured and electroporated in that order. Hence, we cannot say anything about the influence of mRNA electroporation into DCs prior to maturation and can only speculate that immature DCs might be more susceptible for mRNA-mediated signals. However, this should be investigated in separate studies and is beyond the scope of this paper.

Other researchers have indeed observed mRNA-induced maturation of DCs, however under different experimental conditions [19, 26]. Ceppi and coworkers, who worked with porcine monocyte-derived DCs, observed that DC activation can occur after exogenous delivery of mRNA. Lipofection of mRNA induced maturation of immature porcine DCs, that is, MHC class II and CD80/CD86 upregulation [19]. An important element therein is the lipofection-induced production of type I IFN by the DCs, which also showed evidence of maturation. The DC activation was caused by the double-stranded secondary structures formed by the transfected mRNA, and the effect depended on the quantity of lipofected mRNA [19]. It is well established that viral or synthetic double-stranded RNA (dsRNA) acts as a danger signal to DCs, inducing them to produce IFN*α*/*β* and to mature [27]. Furthermore, it was reported that mRNA lipofection has the capacity to activate DCs (human moDCs) [26]. These authors noted the upregulation of activation markers, like CD25, CD80, CD83, CD86, MHC class I, and MHC class II, and cytokine production, like IL-12, IFN*α*, and TNF*α* [26].

Although our different antigen-encoding mRNAs can fold and form dsRNA stretches according to the Mfold Web Server (http://unafold.rna.albany.edu/?q=mfold/RNA-Folding-Form) [28] and thus in theory can also stimulate dsRNA-sensing receptors, we did not observe any DC activation. This can be explained by two main differences of our experiments compared to the experimental setup used by Ceppi et al. and Ni et al.

We used moDCs, which had been matured with a cocktail containing IL-1*β*, TNF*α*, IL-6, and PGE_2_ before the mRNA was introduced, while in the other publications the mRNA was introduced into immature DCs. It might be that the weak stimulus of the introduced mRNA is just not able to change the robust mature phenotype of the cytokine-matured DCs. However, sensing of RNA by receptors should still induce a difference in the transcriptome.

Therefore, it is more plausible that our antigen-encoding RNAs are simply not sensed by the corresponding receptors, because they do not reach the compartments containing these receptors. Indeed it was shown that the RNA must reach the active TLRs in the endolysosomal compartment to be recognized by TLR7, TLR8, and TLR9 and that self-nucleic acids do not enter the TLR-sensing compartment under normal physiological conditions (reviewed in [13]). Only after entering the endolysosomal compartment, the TLRs are activated upon cleavage by resident pH-dependent proteases. This mechanism prevents that self-nucleic acids at different locations in the cell are recognized by the TLRs. Once activated, the TLRs themselves cannot distinguish between foreign and self-nucleic acids; however, the latter do not encounter the active receptors [29]. Normal “naked” mRNA is rapidly degraded by endolysosomal RNas before the receptor is activated. However, if the RNA is protected by lipids, which is the case with lipofection in the publications of Ceppi et al. and Ni et al., or stabilized by protamine [30], or protected by virus particles (reviewed in [13]), it is stable enough to enter the endolysosomal compartment where it is recognized by the activated TLRs resulting in an activation of the DCs.

## 5. Conclusion

Taken together, our data show that electroporation of mature monocyte-derived DCs with antigen-encoding RNA does not deliver a danger signal to the DCs and does not result in a change of the DCs. This is important knowledge for the scientific community using these DCs in vaccination trials, where a stable and robust cell type is needed.

## Figures and Tables

**Table 1:** mRNAs used for transfection.

Antigen	Description	Abbreviation
MelanA	MelanA (MART1) full length wild type	MelanA
NRAS	NRAS-fragment 40 AA around mutation site 61 with DC-LAMP signal and flag-tag	NRAS-DCL
NRAS Q61K	NRAS-fragment 40 AA around mutation Q61K with DC-LAMP signal and flag-tag	NRAS-DCL K
NRAS Q61R	NRAS-fragment 40 AA around mutation Q61R with DC-LAMP signal and flag-tag	NRAS-DCL R
BRAF	BRAF-fragment 67 AA around mutation site 600 with flag-tag	BRAF
BRAF V600E	BRAF-fragment 67 AA around mutation V600E with flag-tag	BRAF E
BRAF	BRAF-fragment 67 AA around mutation site 600 with DC-LAMP signal and flag-tag	BRAF-DCL
BRAF V600E	BRAF-fragment 67 AA around mutation V600E with DC-LAMP signal and flag-tag	BRAF-DCL E
GNAQ	GNAQ-fragment 47 AA around mutation site 209 with DC-LAMP signal and flag-tag	GQ-DCL
GNAQ Q209P	GNAQ-fragment 47 AA around mutation Q209P with DC-LAMP signal and flag-tag	GQ-DCL P
GNAQ Q209L	GNAQ-fragment 47 AA around mutation Q209L with DC-LAMP signal and flag-tag	GQ-DCL L
GNA11	GNA11-fragment 47 AA around mutation site 209 with DC-LAMP signal and flag-tag	G11-DCL
GNA11 Q209P	GNA11-fragment 47 AA around mutation Q209P with DC-LAMP signal and flag-tag	G11-DCL P
GNA11 Q209L	GNA11-fragment 47 AA around mutation Q209L with DC-LAMP signal and flag-tag	G11-DCL L
WT-1	Wilms tumor 1 full length wild type	WT1
GNAQ	Wilms tumor 1 full length with DC-LAMP signal and flag-tag	WT1-DCL
*IKKβ* ^1^	Constitutively active stabilized IKK*β* to activate NF-*κ*B	IKK*β*

^1^Not a tumor antigen, but a DC-activating protein.
